# Harnessing phytochemicals for engineering health solutions

**DOI:** 10.1186/s40246-025-00882-y

**Published:** 2026-01-22

**Authors:** Jenny Ji Hyun Kim, Tyler B. Yang, Xinyue Zhang, Xiangping Lin, Sushma Naranappa Salethoor, Maanasi Madhavan Menon, Michael P. Snyder

**Affiliations:** 1 Department of Genetics, Stanford University, Stanford, CA 94305 USA; 2 Department of Epidemiology and Population Health, Stanford University, Stanford, CA 94305 USA; 3 Stanford Cardiovascular Institute, Stanford University, Stanford, CA 94305 USA; 4 Amrita School of Ayurveda, Amrita Vishwa Vidyapeetham, Amritapuri, Kerala India

**Keywords:** Beneficial exposome, Genome-exposome interactions, Phytochemicals, Multi-omics integration, Precision environmental health, Nature-based interventions, Environmental health

## Abstract

The interaction between the genome and the exposome is increasingly recognized as central to human health and disease. While exposome research has generally focused on adverse exposures such as pollutants and toxins, the concept of the *beneficial exposome*—positive environmental exposures that promote health—remains underexplored. Among the most promising beneficial exposures are plant-derived phytochemicals, a rich class of bioactive compounds with therapeutic potential. Phytoncides, a specific subset of volatile organic compounds released by plants, exemplify this beneficial potential through their antimicrobial, anti-inflammatory, antioxidant, and neuroprotective effects. Historically utilized in traditional medicine across cultures, plant-based remedies containing these compounds are now being examined through modern genomics, exposomics, and systems biology approaches to understand the specific contributions of phytoncides and other bioactive constituents. Emerging data suggest that phytochemicals modulate gene expression, immune function, and metabolic pathways across multiple organ systems, contributing to immune, neurological, endocrine, cardiovascular, respiratory, integumentary, and mental health improvements. However, the evidence base is predominantly preclinical, with limited human validation, considerable heterogeneity in plant-extract composition, and incompletely characterized molecular mechanisms. This review synthesizes current evidence on genome-exposome interactions (GxE) related to plant-derived compounds, highlighting recent mechanistic insights and exploring translational applications—including forest bathing, green space integration in urban design, and bioengineering approaches—while addressing the challenges of clinical translation. As environmental change accelerates, understanding beneficial GxE offers new opportunities for preventative and precision public health interventions and calls for integrating nature-based solutions into modern healthcare paradigms.

## Background

Plants are foundational to both the biosphere and human health, functioning as primary producers in the ecosystem and synthesizing a diverse array of bioactive molecules. Among these are secondary metabolites, including volatile organic compounds (VOCs) such as terpenes (e.g., α-pinene, limonene) and phenylpropanoids (e.g., eugenol, cinnamaldehyde), which exert diverse biological effects in humans [1–3]. These VOCs serve essential roles in plant ecology, mediating inter-plant communication, deterring herbivores, and conferring antimicrobial, antiviral, and insecticidal defense mechanisms. Beyond VOCs, plants also produce non-volatile phytochemicals such as polyphenols, carotenoids, and alkaloids, which are typically ingested rather than inhaled and exert their effects through distinct metabolic pathways.

For millennia, humans have harnessed plant-derived compounds in traditional medical systems such as Ayurveda and Traditional Chinese Medicine (TCM), recognizing their therapeutic potential across a wide range of conditions [4–6]. Traditional practices employ plant-based remedies through both external and internal therapies. External applications include skin treatments, fumigation, and aromatherapy, while internal therapies involve oral, nasal, or rectal administration. A variety of plant parts—including leaves, bark, fruits, flowers, seeds, roots, and sometimes entire plants—are used as medicinal materials to treat diverse ailments. In modern pharmaceutical science, many medicinal compounds have been isolated from plants and served either as direct therapeutics or as structural templates for drug development. Similarly, dietary supplements and nutraceuticals frequently incorporate purified or blended plant-derived ingredients. Recent advances in analytical chemistry, molecular biology, and systems pharmacology have enabled more precise identification and mechanistic characterization of these phytochemicals, elucidating their roles as antioxidants, anti-inflammatory agents, immune modulators, and neuroprotectants [2]. A growing body of evidence supports the therapeutic potential of volatile terpenes such as limonene (from citrus) and pinene (from pine and sage), which have been specifically studied for their anti-inflammatory, antioxidant, and neuroprotective activities [7–11].

The emergence of the exposome concept has transformed our understanding of how environmental factors—including health-promoting exposures such as plant-derived compounds—interact with the human genome to influence health trajectories across the lifespan. The exposome encompasses the totality of environmental exposures an individual experiences, including both external factors (e.g., diet, air pollution, psychosocial stressors) and internal exposures (e.g. hormones, inflammatory mediators, and microbiome-derived molecules). These exposures trigger a complex network of biological responses, such as changes in gene expression, epigenetic modifications, immune regulation, and metabolic activity [12, 13].

However, despite its originally agnostic intent, exposome research has disproportionately focused on adverse exposures and disease risk. In response, a growing number of researchers have advocated for reframing the exposome through a salutogenic lens—one that emphasizes the positive physical, chemical, and psychosocial exposures that promote resilience, healthy aging, and well-being [14, 15].

In alignment with Dr. Christopher Wild’s original vision of exposome research as a prevention-oriented, burden-reducing paradigm [12], we propose a formal conceptual expansion: the “Beneficial Exposome”, or “Beneficial Environmental Exposome.” This framework shifts focus towards the systematic identification and characterization of health-promoting exposures with measurable physiological benefits. This approach is particularly well-suited to studying phytochemicals, as it enables integrative, systems-level investigation of how bioactive plant-derived molecules engage multi-omic networks to modulate human physiology, reduce disease susceptibility, and support well-being.

Gene–environment interactions (GxE) are central to this interplay; individuals with different genetic backgrounds may exhibit differential physiological responses to the same phytochemical exposure, while identical genotypes may lead to divergent health outcomes depending on environmental context. In recent years, research using acute in vivo exposures and in vitro studies has increasingly demonstrated that plant-derived VOCs, specifically phytoncides, modulate key biological processes. However, these exposures have not yet been systematically evaluated through a GxE framework. This represents a significant opportunity, as genetic variants such as single nucleotide polymorphisms (SNPs) can modify physiological responses to environmental exposures, as demonstrated by well-characterized interactions such as pesticide exposure and Parkinson’s disease risk [16, 17]. Future research could build on this foundation by investigating how individual genotypes, or even epigenetic adaptations resulting from multigenerational exposures to forest environments, may influence physiological responsiveness to these compounds. For example, populations living near or frequently exposed to Japan’s hinoki cypress (*Chamaecyparis obtusa*) forests or hinoki-built environments (temples, saunas, furniture) may exhibit distinct biological responses to hinoki exposure, shaped by their environmental and cultural proximity to these phytoncide-rich ecosystems.

In this review, we synthesize current evidence on the health-promoting properties of phytochemicals, with particular emphasis on phytoncides and related plant-derived VOCs. We examine their molecular mechanisms across key human organ systems and highlight emerging mechanistic insights that lay the groundwork for exploring GxE. Importantly, emerging studies suggest that exposures to plant-derived VOCs through practices such as forest bathing, proximity to green spaces, and urban biodiversity may offer accessible and scalable public health interventions to enhance population well-being. By integrating perspectives from traditional medical systems with advances in omics technologies and systems biology, we propose a conceptual framework in which the genome-exposome interface harnesses nature’s chemical diversity to support human health and resilience.

**Fig. 1:**
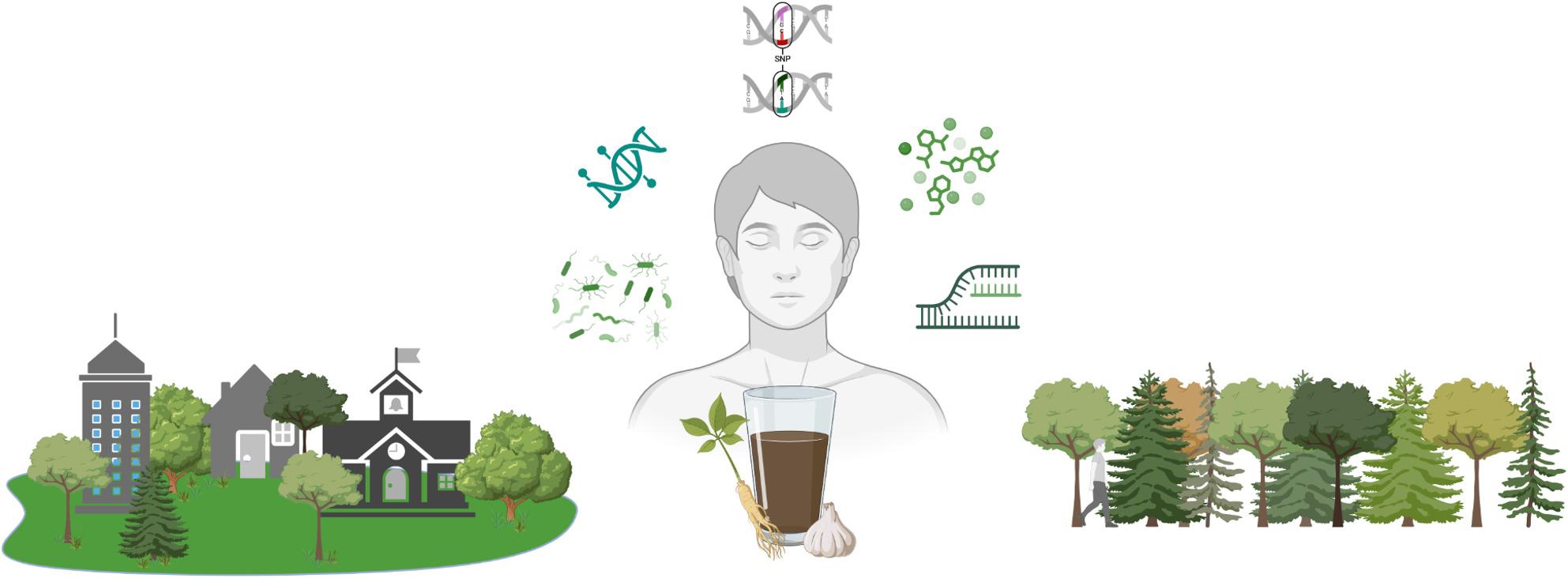
Gene-environment interactions influenced by plant-based exposures across molecular layers

## Traditional uses of plant compounds

### Historical context

The use of medicinal plants for health and healing dates back thousands of years and spans nearly every culture. Across ancient civilizations—from Egypt and Greece to China and India—scholars systematically recorded the therapeutic uses of local plants, forming the foundation of many traditional medical systems [18, 19].

Medicinal plant components have been prepared in a variety of dosage forms, including decoctions, distillates, powders, fresh juices, pastes, fermented liquids, and both hot and cold infusions. Additionally, herbal ingredients are often infused in fat-based media such as oil, ghee, or milk. These dosage forms were prepared using single herbs or combinations of multiple herbs, integrating their therapeutic components to create a multifaceted approach in addressing various aspects of a disease. Traditional practices also incorporate aromatic therapies, such as moxibustion and essential oil (EO) inhalation, which leverage plant VOCs. These ethnobotanical traditions laid the groundwork for both empirical and systematic approaches to traditional medicine [2].

Ayurveda, the traditional system of medicine in India, exemplifies this comprehensive approach through a vast pharmacopoeia of plant extracts, often as complex polyherbal formulations [20]. For example, ashwagandha (*Withania somnifera)* is used as an adaptogen to reduce stress and enhance vitality, while turmeric (*Curcuma longa)* is prized for its anti-inflammatory and antioxidant effects, largely attributed to the polyphenolic compound curcumin [21–23].

Similarly, TCM utilizes combinatory approaches, blending multiple herbs that work in combination to target illnesses while optimizing dose and delivery of bioactive compounds [24, 25]. TCM operates on the principle of “*Jun-Chen-Zuo-Shi*,” a hierarchical framework that guides multi-component formulations. In this system, “*Jun”* (emperor) is the primary ingredient that directly targets the disease; “*Chen*” (minister) supports Jun by enhancing its therapeutic effect; “*Zuo*” (adjuvant) further boosts efficacy and mitigates potential side effects; and “*Shi”* (messenger) balances the action of all other ingredients [24]. Examples include *Ginkgo biloba*, commonly used for cognitive support and neuroprotection [26, 27], and *Panax ginseng*, known for its anti-inflammatory and anti-diabetic properties, as well as its ability to enhance cognitive performance [28, 29]. A study examining their combination found enhanced cognitive effects compared to individual administration [30]. The interactive nature of traditional formulations is exemplified by curcumin, whose bioavailability is markedly increased when co-administered with piperine, an alkaloid from black pepper (*Piper nigrum*), demonstrating how phytochemical interactions can enhance therapeutic efficacy [31].

Other global traditions further demonstrate this universal recognition of plant therapeutics. These include eucalyptus and tea tree oil use by Australian Native peoples for wound healing and infection [32], and the use of sage, cedar, and pine in Native American spiritual and medicinal practices. In Japan, hinoki cypress holds significant cultural and spiritual value; its antimicrobial, aromatic, and durable properties have been harnessed in temple architecture for centuries. Traditional South Korean practices have long utilized the bark of the hinoki tree for its perceived healing properties, particularly in supporting cancer patients. Emerging research now suggests that hinoki-derived compounds may indeed exhibit anti-inflammatory and anti-cancer activities [33–36].

These diverse traditional systems exemplify holistic, individualized approaches to health that contrast with the Western biomedical model of “one-drug, one-target, one disease,” which focuses on isolated compounds with well-defined mechanisms of action. Traditional formulations’ use of complex mixtures derived from single or multiple herbs presents challenges for mechanistic studies and standardization. Each part of a medicinal plant may contain distinct active compounds that offer multiple therapeutic effects. However, extracting and concentrating individual phytochemicals outside their native context can raise important safety concerns. Some compounds that are safe and effective within whole-plant preparations may become toxic or poorly tolerated when administered in purified form.

This principle is illustrated by reserpine, an alkaloid extracted from sarpagandha (*Rauwolfia serpentina*). While historically used for hypertension and neuropsychiatric conditions, it has been associated with side effects including lethargy, sedation, depression, and other psychiatric symptoms when used at doses above 0.5 mg/day [37]. In contrast, traditional use of the whole plant has been reported to produce fewer side effects, likely due to the presence of accompanying phytochemicals that modulate reserpine’s activity, resulting in a more balanced therapeutic profile [38].

### Transition to modern science and regulation

Modern pharmacology has successfully isolated numerous plant-derived compounds that now serve as cornerstone therapies, such as aspirin (from willow bark) for pain relief, artemisinin (from *Artemisia annua L.*) as an antimalarial agent, and paclitaxel (from the Pacific yew tree) for cancer chemotherapy [39–42]. However, despite these successes, the path from traditional use to clinical approval remains complex. In the US, most herbal extracts fall under the Dietary Supplement Health and Education Act (DSHEA) of 1994 [43, 44], which allows consumer access but offers limited physician guidance due to gaps in data on toxicity, efficacy, and standardized dosing. Compounding this challenge, EOs used in aromatherapy are regulated as cosmetics or fragrances under the U.S. Food and Drug Administration (FDA) [45], further restricting therapeutic claims and limiting integration into clinical practice [46].

The development of traditional plant-based therapies into modern botanical drugs faces several key barriers: ingredient standardization, quality control, and inconsistent regulatory frameworks across countries [47–50]. The drug development pipeline—from compound discovery and preclinical testing through Investigational New Drug (IND) applications, clinical trials, and regulatory approval through New Drug Applications (NDAs)—is both lengthy and resource-intensive [51, 52].

These challenges are magnified by the inherent variability of plant chemistry. Even within a single species, phytochemical composition can vary significantly depending on factors such as geography, growth conditions (including biotic and abiotic stressors), soil nutrients, climate, time of harvest, and extraction methods [45, 47, 53]. This variability presents unique standardization challenges that differ fundamentally from conventional pharmaceutical development.

To support the safe and effective clinical use of EOs and other plant-derived products, developing specific regulatory guidelines and quality standards that encompass proper cultivation, manufacturing, labeling, and quality control is essential [54].

### Contemporary integration and global trends

Growing consumer interest in natural therapies, coupled with advances in analytical techniques, has sparked renewed scientific attention to plant-based medicine [47, 55–58]. Nature-based interventions like the Japanese practice of Shinrin-yoku (forest bathing) and aromatherapy are increasingly recognized globally for their physiological benefits. Recent studies have demonstrated that forest VOC exposure can reduce blood pressure, lower stress hormones, and enhance immune function [34, 59–62], with ongoing studies now beginning to elucidate underlying molecular and genomic mechanisms.

This scientific validation comes at a time when traditional medicine remains central to global healthcare delivery. The World Health Organization (WHO) estimates that up to 80% of the world’s population relies on traditional medicine for primary health care [63, 64]. Several countries have formalized integration efforts: China has implemented national policies supporting TCM, India has established the Ministry of AYUSH (Ayurveda, Yoga & Naturopathy, Unani, Siddha and Homeopathy), and the WHO has launched a global traditional medicine strategy [56, 65–67].

These efforts align with broader international frameworks recognizing the interconnectedness of health systems. The One Health Joint Plan of Action of the Quadripartite Organizations—the Food and Agriculture Organization of the United Nations (FAO), the United Nations Environmental Programme (UNEP), the World Organisation for Animal Health (WOAH), and the WHO—emphasizes the interconnectedness of human, animal, plant, and environmental health [68]. This framework underscores the importance of preserving biodiversity for both ecological balance and human well-being.

Moving forward, integrating traditional medicine into modern healthcare systems will require harmonizing traditional practices with contemporary scientific validation, potentially enhancing global health outcomes and offering more holistic treatment options.

### Challenges and opportunities

Despite the rich history and widespread use of traditional medicine systems, a critical need remains for rigorous in vitro, in vivo, and clinical studies to validate the safety, efficacy, and mechanisms of action of plant-derived compounds, including multi-herb formulations. This evidence is essential for understanding potential side effects, determining optimal dosages, and enabling their integration into evidence-based medicine.

Beyond the regulatory and standardization challenges previously discussed, a key opportunity lies in leveraging emerging technologies to accelerate translation. Artificial intelligence (AI), high-throughput screening, and advanced omics platforms offer powerful tools for identifying, characterizing, and optimizing bioactive plant compounds within a systems-level context [45, 69]. These tools can also support evidence-based classification of complex herbal formulations and contribute to designing targeted, personalized interventions.

The scale of this opportunity is remarkable. While an estimated 250,000 to 500,000 higher plant species exist, only 5–6% have been screened for bioactivity, leaving vast untapped potential for therapeutic discovery [70, 71]. Additionally, traditional medical systems offer valuable starting points for hypothesis generation, as many have long incorporated individualized assessment based on observable phenotypes, lifestyle factors, and dietary patterns. These personalized approaches mirror key principles of modern precision medicine [49, 72, 73].

Perhaps most promising is the potential to understand the GxE underlying traditional practices. This approach aligned with precision environmental health [74], may unlock novel strategies for prevention and treatment in a rapidly changing world, while also facilitating broader integration into Western medical paradigms.

## Bioactive phytochemicals: classes and characterization

### Phytoncides and other major phytochemical classes

Building on the diverse array of plant secondary metabolites previously discussed, phytoncides represent a distinct subgroup of plant-derived VOCs, first described by Boris Tokin in the late 1920s. Since their discovery, phytoncides have attracted considerable scientific interest due to their ecological significance and potential biomedical applications [34]. These volatile compounds serve multiple ecological functions, including chemical defense against herbivores and pathogens, attraction of pollinators, and mediation of environmental stress [75, 76].

Among phytoncides, terpenes represent the most extensively studied chemical class, encompassing monoterpenes (including α-pinene, β-pinene, limonene, myrcene, and camphene) and sesquiterpenes (such as β-caryophyllene and humulene). These volatile compounds are particularly abundant in coniferous forests, citrus fruits, and aromatic herbs [77, 78]. Beyond terpenes, other prominent phytochemical classes include phenylpropanoids (such as eugenol from clove and cinnamaldehyde from cinnamon) and oxygenated terpenoids (such as linalool from lavender and citral from lemongrass) [79, 80].

While phytoncides focus specifically on volatile compounds, the broader phytochemical landscape also encompasses non-volatile bioactive molecules, including polyphenols, carotenoids, and alkaloids, which contribute to plant therapeutic potential through different exposure pathways. The functional diversity of phytoncides and related bioactive metabolites reflects the evolutionary pressures plants face in their natural environments.

### Sampling, extraction, and characterization of phytochemicals

Accurate sampling, extraction, and characterization of plant-derived phytochemicals are essential for both research and therapeutic applications.

In field studies, plant-derived VOCs, including phytoncides, are commonly collected using adsorption-based methods such as activated carbon or polymeric sorbents, which effectively trap volatile molecules for subsequent analysis [81, 82]. Headspace sampling techniques, both static and dynamic, have significantly advanced the study of plant volatiles by enabling non-destructive, rapid, and sensitive detection. Among these, solid-phase microextraction (SPME) has emerged as a particularly valuable method due to its simplicity, high sensitivity, and ability to detect trace-level concentrations of VOCs [83, 84].

For plant tissues, once sampled, extraction methods play a critical role in isolating phytochemicals. Steam distillation remains the most widely used technique for obtaining EOs and volatile fractions from leaves, flowers, and woody tissues [85]. For example, EO from hinoki cypress is traditionally produced via steam distillation, yielding a complex mixture rich in terpenes and volatile alcohols [86].

For broader phytochemical recovery, solvent-based extraction methods employing ethanol, hexane, methanol, or acetone expand the spectrum of recoverable compounds, particularly those with lower volatility or higher polarity, such as flavonoids, phenolic acids, and alkaloids [87, 88]. More recently, supercritical fluid extraction (SFE) using carbon dioxide (CO₂) has gained popularity due to its ability to preserve heat-sensitive compounds, achieve high extraction efficiencies, and minimize solvent residues, offering a greener alternative to conventional solvent extraction [89, 90].

Following sampling and extraction, gas chromatography–mass spectrometry (GC-MS) remains the principal analytical platform for identifying and quantifying volatile constituents due to its high sensitivity, resolution, and extensive spectral libraries [91, 92]. For non-volatile compounds, high-performance liquid chromatography (HPLC), often coupled with diode-array detection (DAD) or mass spectrometry (LC-MS), is routinely employed [93, 94]. Nuclear magnetic resonance (NMR) spectroscopy complements these chromatographic techniques by providing detailed structural elucidation and confirmation of novel or complex phytochemical structures (95,96).

Recent methodological advances in the sampling, extraction, and characterization of phytochemicals have markedly improved our ability to map the chemical diversity of botanicals and relate it to potential health benefits. Yet, the full realization of this potential hinges on overcoming data integration challenges that stem from complex plant matrices, limited biological endpoints, and intricate regulatory pathways. A concerted effort combining optimized protocols (e.g., validated analytical protocols, documentation of cultivation conditions, storage requirements), standardization initiatives (e.g., adoption of standardized protocols encompassing sampling, extraction, analysis, and reporting) as further detailed in the Sect. 6.2.3, and rigorous clinical validation will be necessary for plant-derived phytochemicals to become robust, standardized, and trusted components of therapeutic strategies.

## Genome–exposome interactions of plant-derived compounds

### The exposome framework

The exposome concept, coined twenty years ago by Dr. Christopher Wild, encompasses all exposures across one’s lifetime [12]. It complements the genome, representing everything that the genome doesn’t cover, and holds great potential to uncover the non-genetic factors in complex diseases and GxE [97, 98]. These exposures, ranging from air, water, soil, and greenspace to climate, diet, and socioeconomic status, collectively influence human health.

Plants represent a unique and multi-faceted component of the human exposome through multiple exposure pathways and mechanisms [99]. They function both as modulators of environmental exposures and as direct sources of bioactive compounds. Plants can reduce indoor air pollutants, such as formaldehyde and acetone [100–102], while soil conditions and environmental factors influence plant phytochemical composition, affecting downstream human exposure through dietary and inhalation pathways [103]. Additionally, plants contribute to environmental regulation through temperature and humidity control, soil stabilization, and nutrient cycling [104]. While plant VOCs serve ecological functions such as improving stress resistance and regulating growth, increasing evidence highlights their potential benefits for human health, including antioxidative, anti-inflammatory, and cognitive enhancing properties, as detailed in Sect. 5.

### Omics approaches

Recent advancement in omics technologies have brought new insights into GxE. Multi-omics approaches—including genomics, transcriptomics, proteomics, epigenomics, lipidomics, and metabolomics, and metagenomics—have been independently and collectively employed to delineate molecular signatures associated with environmental exposures that modulate disease progression trajectories.

These approaches have revealed diverse exposure-response patterns across biological systems. Transcriptomic analyses have identified gene expression signatures associated with exposure to a range of environmental chemicals, implicating key biological pathways involved in inflammation, oxidative stress, DNA damage, and apoptosis [105]. Similarly, mass spectrometry (MS)-based proteomics has been utilized to characterize the impact of environmental exposures on protein expression and post-translational modifications, including phosphorylation, acetylation, and ubiquitination [106]. Complementing these approaches, MS-based lipidomic profiling has been applied to investigate exposure effects on bioactive lipid mediators in human airways [107], while metabolomic investigations have detected exposure-derived metabolites and their associations with alterations in endogenous metabolic pathways linked to various toxicological outcomes including immunosuppression, hepatotoxicity, and nephrotoxicity [108].

The true power of these technologies emerge through integration. Multi-omic integration creates comprehensive biological profiles [109], as demonstrated by several landmark studies. Research utilizing a multi-omic network to profile individual external exposures and internal molecular changes revealed significant, dynamic associations with pathways related to immune, kidney, and liver function. This work demonstrated the exposome’s potential impact on personalized health assessment. Another innovative approach employed frequent blood microsampling to analyze thousands of metabolites, lipids, cytokines, and proteins using MS-based multi-omics combined with continuous physiological data from wearable sensors. This approach revealed individualized inflammatory and metabolic responses to dietary interventions and uncovered extensive molecular fluctuations associated with intra-day physiological changes—such as heart rate, glucose, and cortisol levels—and physical activity [110]. Similarly, aging research has benefited from multi-omics approaches; a recent study uncovered nonlinear molecular trajectories during aging, identifying two key inflection points around ages 44 and 60 that correspond to coordinated dysregulation across immune, metabolic, and cardiovascular pathways [111].

For plant-derived compound research specifically, incorporating multi-omics approaches to study GxE can greatly facilitate the identification and quantification of exposures and their corresponding biological effects. This is particularly relevant for understanding how genetic variation influences individual responses to phytochemical exposures, a critical gap in current research.

Despite their promise, multi-omics approaches face several practical limitations. While costs and turnaround times have rapidly decreased, these approaches still require highly specialized personnel, equipment, and substantial financial investment. The “big data” generated is often heterogeneous, requiring rigorous statistical tools for analysis and interpretation. These complex datasets also require robust computational infrastructures for modeling and storage, in addition to data security and privacy protection measures. As new technologies like wearable sensors continue to emerge, developing standardized protocols for data collection and sharing becomes increasingly important for better integration and reproducibility [112, 113].

## Influence of plants on human health

Here, we highlight the effects of plant-derived compounds, with specific emphasis on VOCs, across six major human systems: immune, neurological, endocrine, cardiovascular, respiratory, and integumentary, as well as their broader impact on mental health. These domains represent key physiological axes through which environmental exposures interface with genomic regulation and systemic responses. In the following subsections, we examine each system and highlight existing research demonstrating the potential of phytoncides to modulate physiological function and support health. Each subsection concludes with an example of a traditional medical practice that employs plant-derived compounds to address acute or chronic conditions relevant to that system.

**Fig. 2:**
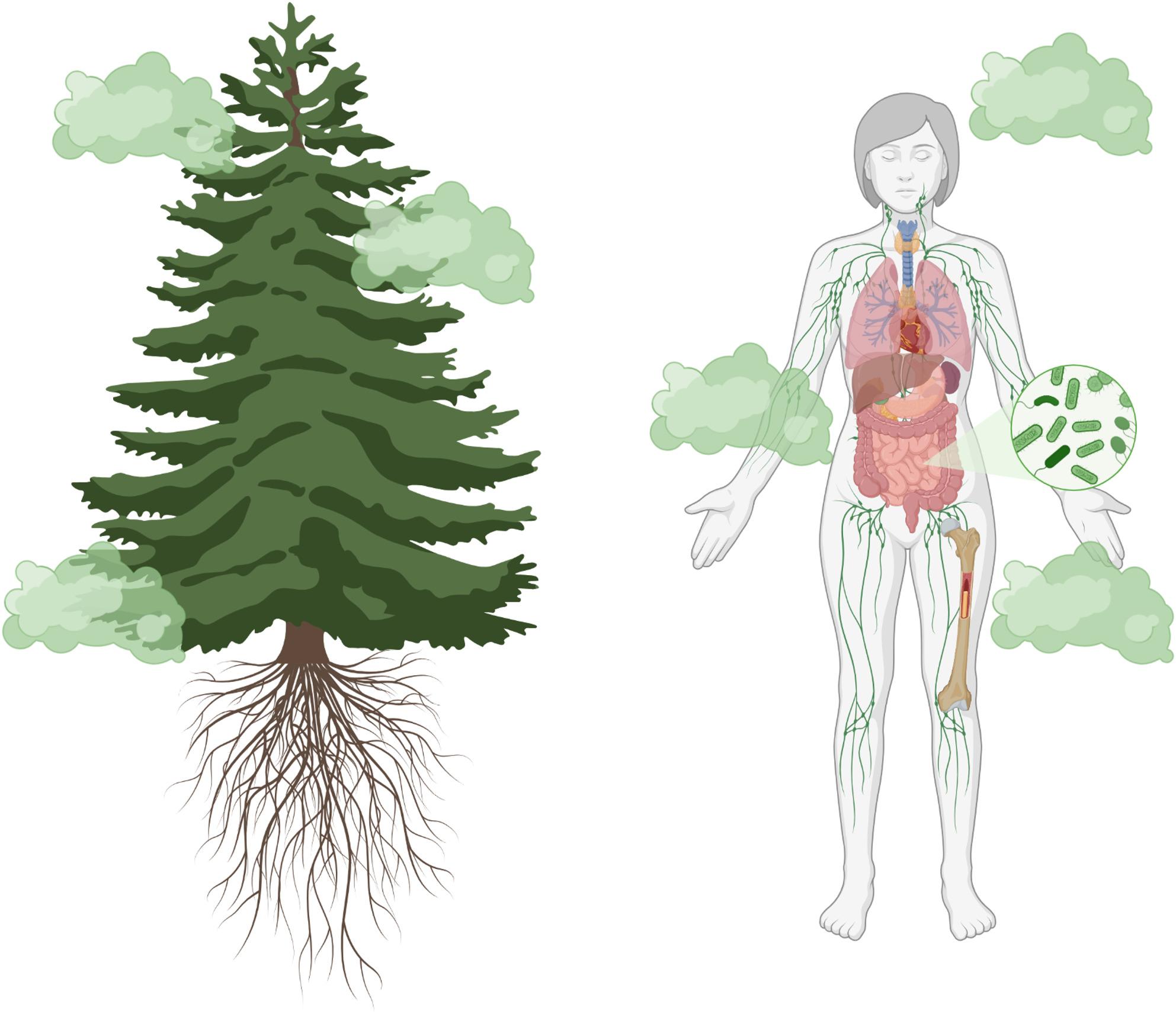
Phytoncide emission from plants and their impact on human organ systems

### Immune system

Plants and their bioactive compounds have long been recognized for their ability to modulate immune responses. Recent research highlights the immunomodulatory, anti-inflammatory, and cytoprotective properties of plant-derived terpenes and phenolics.

α-Pinene represents one of the most well-studied immunomodulatory compounds. This volatile compound found in pine trees and other conifers has been shown to enhance natural killer (NK) cell activity. NK cells are lymphocytes of the innate immune system that identify and eliminate virus-infected or cancerous cells. In an in vitro assay, human NK-92mi cells treated with α-pinene exhibited upregulation of surface markers (CD56, CD107a) and increased expression of cytolytic effectors (perforin, granzyme B), with effects consistent with activation of the ERK/AKT signaling pathways. In vivo, a mouse allograft model using CT-26 colon cancer cells showed that α-pinene treatment (40 mg/kg) reduced tumor burden by 42.83% [114]. Complementary forest bathing studies in human subjects reported increased NK cell numbers and activity, lasting more than seven days following exposure to phytoncide-rich environments (α-pinene, β-pinene detected in forest air but not city air), suggesting sustained immunological effects from phytoncide exposure [62, 115].

Beyond immune surveillance, α-pinene also exhibits anti-inflammatory properties. An in vitro study in mouse peritoneal macrophages demonstrated that α-pinene attenuated lipopolysaccharide (LPS)-induced inflammatory responses by downregulating pro-inflammatory mediators including IL-6, TNF-α, inducible nitric oxide synthase (iNOS), and COX-2 through MAPK and NF-κB pathway inhibition [9]. This dual action—immune stimulation and inflammation suppression—positions α-pinene as a promising candidate for both immunotherapeutic and anti-inflammatory applications.

Linalool, another compound of interest, is a monoterpene alcohol found in lavender, coriander, and members of the *Plantaginaceae* family. It has demonstrated cytotoxic and immunoregulatory properties. In vitro, linalool induced apoptosis in multiple cancer cell lines promoted cytokine release—including IFN-γ, IL-2, and TNF-α—indicative of a T helper 1 (Th1)-type immune response [116]. In vivo studies showed efficacy in improving healing outcomes and decreasing inflammatory infiltration in methicillin-resistant *Staphylococcus aureus* (MRSA)-infected mice, while also promoting microbial diversity on the skin surface [117]. Additionally, in a carrageenin-induced paw edema model in rats, linalool reduced inflammation following systemic administration [118]. These findings suggest that linalool supports both immune resolution and epithelial homeostasis during infection.

D-limonene, a cyclic monoterpene found in citrus EOs, influences adaptive immunity through different mechanisms. Ex vivo studies using murine T lymphocytes demonstrated that d-limonene and its metabolites (limonene-1–2-diol, perillic acid) suppressed cytokine production (IFN-γ, IL-2, TNF-α, IL-4, IL-13) and downregulated activation markers (CD25, CD69, CD40L) on CD4 + and CD8 + T cells, with cytotoxic effects observed only at higher concentrations [119].

Eucalyptol (1,8-cineole), found in eucalyptus, demonstrates dose-dependent effects on respiratory immune responses. In a three-week rat study, low-to-moderate doses (30–100 mg/kg) upregulated CD8 + T-cell activity in the lungs without impairing macrophage function. However, high doses (300 mg/kg) suppressed CD8 + activity and macrophage phagocytosis while also decreasing circulating B cells, NK cells, and IgA levels [120]. At moderate doses, 1,8-cineole may enhance mucosal and cellular immunity; at high doses, it could compromise immune defenses.

Carvacrol, commonly found in oregano and thyme, targets inflammatory pathways directly. In a broiler chicken model following LPS-induced inflammation, oral carvacrol administration significantly reduced expression of pro-inflammatory cytokine genes (TNF-α, IL-1β, IL-6) as well as TLR4 and NF-κB p65 signaling molecules. Carvacrol also reduced avian β-defensin-9 (AVBD-9) [121].

Finally, β-Caryophyllene (BCP), found in cinnamon, cloves, rosemary, and black pepper, demonstrates broad pharmacological effects, including antioxidant and antimicrobial activity. A comprehensive review of BCP studies indicates its anti-inflammatory effects occur mainly through cannabinoid receptor 2 (CB2) activation and the peroxisome proliferator-activated receptor (PPAR)γ pathway. BCP suppressed both protein and mRNA expression of IL-6 and other pro-inflammatory cytokines, while increasing the anti-inflammatory cytokine IL-13. Its antioxidant effects involve activation of the Nrf2/HO-1 antioxidant axis, inhibition of 3-hydroxy-3-methylglutaryl-coenzyme A (HMG-CoA) reductase activity, and reductions in oxidative stress biomarkers [122].

#### Traditional applications

Traditional medicine systems have long relied on aromatic spices rich in EOs—such as cumin, clove, turmeric, ginger, and mint—for immune system support. These spices are commonly administered both as dietary components and as medicinal preparations. For example, ginger is rich in 6-gingerol, a bioactive compound with anti-inflammatory and immunomodulatory effects mediated through inhibition of NF-κB and protein kinase C-α (PKC-α) signaling pathways, which suppresses iNOS and TNF-α expression while reducing reactive oxygen species (ROS) production. Additionally, 6-gingerol demonstrates anti-angiogenic activity by inhibiting VEGF- and bFGF-induced endothelial cell proliferation, contributing to reduced cell metastasis in preclinical models [123–125].

### Neurological system

#### Cognitive function

A growing body of evidence suggests that phytoncides and plant-derived EOs can influence cognitive performance through diverse neurobiological mechanisms. These compounds appear to modulate neurotransmission, oxidative stress, and cortical activation patterns, leading to measurable effects on attention, memory, and executive function.

Peppermint EO exemplifies these cognitive effects. Rich in menthol and menthone, it has demonstrated cognitive-enhancing effects in both in vitro and in vivo studies. In vitro, it inhibits acetylcholinesterase (AChE) and modulates GABA_A_ and nicotinic receptors, increasing calcium mobilization in neurons—mechanisms associated with enhanced synaptic activity. In a double-blind, placebo-controlled, balanced cross-over trial, a single dose of peppermint EO enhanced working memory and sustained attention in young adults while also reducing mental fatigue during cognitively demanding tasks [126].

Phytoncides also demonstrate measurable neurophysiological effects. In a randomized, double-blind, controlled study, electroencephalogram (EEG) recordings showed that brief exposure to phytoncide odor reduced β-band activity in the occipital and parietal cortices—an effect the authors interpret as relief from anxiety, depression, and stress symptoms common in patients with mild cognitive impairment [127]. Supporting these findings, older adults with mild cognitive impairment exhibited improved executive function, as measured by the Stroop task, following phytoncide inhalation. This was accompanied by reduced prefrontal cortex activation on functional near-infrared spectroscopy (fNIRS), suggesting that phytoncides may reduce compensatory overactivation and restore neural efficiency [128].

Multiple EO constituents contribute to these cognitive benefits. For example, an in vivo and in vitro study using a Wistar rat model of Alzheimer’s disease demonstrated that inhalation of arar tree (*Tetraclinis articulata)* EO reversed memory deficits and oxidative stress markers by normalizing pathologically elevated hippocampal AChE activity and enhancing key antioxidant enzymes—superoxide dismutase (SOD), catalase (CAT), and glutathione peroxidase (GPx) [130].

However, effects are not uniformly beneficial. In a controlled office environment study, lemon EO (*Citrus limonum*) (rich in d-limonene) exposure in human subjects was associated with faster reaction times but reduced inhibitory control and memory sensitivity, suggesting a potential trade-off between processing speed and cognitive accuracy [131]. These mixed effects may stem from differences in compound composition, delivery methods, or study context.

##### Traditional applications

Brahmi (*Bacopa monnieri*), *Ginkgo biloba*, and ashwagandha are commonly used in traditional medicine systems for cognitive support, particularly in conditions like Alzheimer’s disease [132]. A comprehensive review of Brahmi research found that triterpenoid saponins (bacosides) in brahmi restore synaptic activity, enhance neuronal synthesis and kinase activity, and increase neurotransmitter availability, including serotonin [133]. Traditionally, these herbs are administered orally or nasally using fat-based media—such as medicated ghee, oil or milk—as lipids facilitate the transport of active compounds across the blood-brain barrier.

#### The microbiome and the gut-brain axis

Beyond direct neurological effects, EOs can modulate brain function indirectly via the microbiota-gut brain axis (MGBA), a complex bidirectional signaling network involving microbial metabolites, immune signals, and the vagus nerve.

The MGBA represents a central pathway in neuroendocrine and neuroimmune regulation, influencing mood, cognition, inflammation, and systemic homeostasis [134]. Mediated in part by the vagus nerve, this axis serves as the key interface for modulating brain health through gut-derived signals. Given their lipophilic nature, EO constituents can cross biological membranes, including the blood-brain barrier, positioning EOs as promising MGBA modulators [135].

In a controlled in vivo murine study, eucalyptus EO (EEO) administration (10 mg/kg) increased sleep duration by 56.9% and elevated levels of sleep-promoting neurotransmitters (glutamine, GABA, glycine, tryptophan, N-acetylserotonin, and 5-hydroxyindoleacetic acid), while enhancing gut microbial diversity and short-chain fatty acid (SCFA)-producing microbiota. The enrichment of GABA- and glycine-producing microbes suggests a microbial mechanism underlying EEO’s neuromodulatory effects [136].

Similarly, may chang (*Litsea cubeba*) EO demonstrated anti-inflammatory effects, in LPS-induced intestinal inflammation in mice, reducing hepatic and intestinal TNF-α, IL-6, and IL-1β levels alongside dose-dependent shifts in gut microbiota composition (increased *Lactobacillaceae*, *Lachnospiraceae*; decreased *Muribaculaceae*) [137]. In mouse models of Parkinson’s disease, curcumin has demonstrated neuroprotective effects through gut microbiota modulation. Curcumin improved motor deficits, protected dopaminergic neurons, and modulated gut microbiota composition in MPTP-induced PD mice. Key mechanisms include increased tyrosine-dopamine metabolism, reduced α-synuclein aggregation, and decreased neuroinflammation [138–140].

In vitro screening of 13 EOs against 12 gut microbial strains revealed selective antimicrobial activity; carvacrol, cinnamaldehyde, citral, and thymol (and oregano, thyme, rosemary oils) suppressed pathogenic strains (*C. perfringens*,* S. epidermis*,* E.coli*) while sparing beneficial microbes (*Bifidobacterium* spp., *Lactobacillus reuteri*) [141].

Expanding beyond the gut-brain connection, the skin-gut axis has emerged as another important bidirectional communication pathway. The skin serves as a “window” into overall health, with skin microbiome dysbiosis reflecting or potentially influencing gut microbial imbalances [142]. EOs can interact with the skin microbiome via topical application or diffusion [143], suggesting a novel pathway whereby this interaction may affect systemic microbiota.

##### Traditional applications

Traditional Ayurvedic medicine has long recognized connections between the gut and brain function. Several herbs like turmeric, ginger, and triphala (*Emblica officinalis*,* Terminalia bellerica*,* Terminalia chebula*), shankapushpi (*Convolvulus pluricaulis*), jatamansi (*Nardostachys jatamansi*), *Ginkgo biloba*, *Panax ginseng*, mandukaparni (*Centella asiatica*), brahmi, and ashwagandha have been used traditionally to support cognitive function [144, 145]. Emerging evidence primarily from animal models, suggests that some Ayurvedic preparations may influence brain health through gut microbiome modulation, with triphala demonstrating the most well-characterized effects on beneficial gut bacteria and their metabolites. Additionally, Ayurveda employs medicated enemas (basti) as a gastrointestinal route to deliver herbal preparations for treating neurological diseases [146].

#### Pain modulation

Pain perception involves both peripheral and CNS pathways, making it an important target for EO-based interventions. EOs may offer natural analgesic effects through modulation of inflammatory cascades, neurotransmitter receptors, and neurosensory pathways.

Modern pain management often relies on pharmacological analgesics, which, despite their efficacy, are associated with significant side effects. EOs, traditionally used for pain relief in many cultures, represent a promising complementary approach.

For instance, eucalyptus EOs have long been employed in Brazilian folk medicine. In in vivo murine studies, intraperitoneal administration of essential oils from three *Eucalyptus* species (*E. citriodora*,* E. tereticornis*,* E. globulus*) demonstrated both peripheral (reduction of acetic acid-induced writhe) and central (prolonged hot-plate thermal reaction time) analgesic effects. The oils may act via inhibition of arachidonic acid metabolism, reducing prostaglandins and thromboxane synthesis [147].

Clinical studies support EO-based analgesia. A randomized, double-blind, placebo-controlled study of 54 patients reported significant pain reduction after *Piper nigrum* EO inhalation compared to sesame oil placebo [148]. While the mechanism was not investigated in this clinical study, prior preclinical studies have identified β-caryophyllene, a major constituent of *Piper nigrum*, as a selective cannabinoid receptor 2 (CB2) agonist, suggesting that endocannabinoid modulation may contribute to analgesic effects [149].

EO blends have also shown efficacy for gynecological pain. In a randomized, double-blind clinical trial of 48 women with primary dysmenorrhea, topical massage with a blend of lavender (*Lavandula augustifolia)*, clary sage (*Salvia sclarea*), and marjoram (*Origanum majorana*) significantly reduced both menstrual pain severity and duration compared to synthetic fragrance control [150]. The blend contained four key analgesic components: linalyl acetate (36.84%), linalool (22.53%), 1,8-cineole (17.21%), and β-caryophyllene (2.69%). These compounds may contribute to pain relief through anti-inflammatory mechanisms, as 1,8-cineole has been shown to inhibit cytokine production including TNF-α and IL-1β [150, 151].

EOs have also demonstrated perioperative benefits. In a randomized, placebo-controlled trial of 54 patients undergoing laparoscopic adjustable gastric banding (LAGB), lavender oil inhalation significantly reduced postoperative morphine requirements compared to placebo (2.38 mg vs. 42.6 mg, *p* < 0.04), with fewer patients requiring opiod analgesia (46% vs. 82%, *p* = 0.007). No differences were observed in antiemetic or antihypertensive requirements or post-anesthesia care unit discharge times. Notably, lavender patients were significantly less sedated at discharge (*p* = 0.003), suggesting lavender oil provided additive analgesic effects without adverse perioperative consequences [152].

##### Traditional applications

Traditional medicine systems have long relied on aromatic herbs and resins with potent volatile compounds—such as ginger, frankincense (*Boswellia serrata*), turmeric [153], camphor (*Cinnamomum camphora*) [154], and guggul (*Commiphora mukul*) [155])—for analgesic effects. Administration includes topical applications and oral ingestion for pain relief. These compounds reduce inflammation and modulate neurotransmitters and ion channels involved in pain signaling. For example, camphor’s analgesic effects involve inhibition of transient receptor potential ankyrin 1 (TRPA1) channels and desensitization of transient receptor potential vanilloid 1 (TRPV1) receptors. Camphor initially activates TRPV1, followed by rapid and complete desensitization (more so than capsaicin), which may contribute to its analgesic properties [154]. Clinical evidence supports these traditional uses: a meta-analysis of seven clinical trials involving 545 participants demonstrated that frankincense/shallaki effectively reduced pain (VAS, WOMAC pain) and stiffness while enhancing joint mobility in osteoarthritis patients. Among its active constituents, 3-O-Acetyl-11-keto-β-boswellic acid (AKBA) serves as a powerful inhibitor of leukotriene-driven inflammation and 5-lipoxygenase (5-LOX) enzyme activity [156].

### Endocrine system

EOs and phytoncides have demonstrated a variety of effects on the endocrine system, particularly in relation to stress hormone regulation and reproductive hormone modulation. These plant-derived compounds appear to interact with both the central nervous and endocrine systems to influence hormonal balance and autonomic function in humans and in vitro models.

The endocrine-modulating potential of five commonly used EOs—niaouli, orange, tea tree, wintergreen, and ylang-ylang—was investigated in vitro using the JEG-Tox model, which assesses hormone secretion in human placental cells. Researchers measured secretion of four critical pregnancy-related hormones—progesterone, estradiol, human hyperglycosylated chorionic gonadotropin (hCG), and human placental lactogen (hPL)—alongside P2 × 7 receptor activation, a marker of cellular stress. All five EOs significantly altered hormone secretion profiles without inducing cytotoxicity. Niaouli EO elevated progesterone and hCG levels; wintergreen EO increased the secretion of three hormones: progesterone, hCG, and hPL; orange EO elevated estradiol and hPL; and tea tree EO enhanced hPL levels. Notably, the major isolated constituents of these oils (1,8-cineole, limonene, 4-terpineol, methyl salicylate, and benzyl salicylate) often produced weaker or opposing effects compared to the complete oils, suggesting a combinatory “cocktail” effect of minor compounds within whole EOs. These findings indicate that EOs may act as nuanced hormonal modulators rather than classical endocrine disruptors [157].

Cinnamon (*Cinnamomum zeylanicum*) improves glucose metabolism through brain-mediated mechanisms. Eugenol, a major component, enhanced insulin signaling in astrocytes via GSK3 and AKT phosphorylation. In obses diabetic mice, cinnamon extract improved brain insulin sensitivity, reduced blood glucose and hepatic triglycerides, and increased liver glycogen. These hepatic effects appear centrally mediated through the brain-liver axis than direct liver action [158]. Fennel EO demonstrates antidiabetic effects. In streptozotocin-induced diabetic rats, fennel oil (30 mg/kg) corrected hyperglycemia, increased glutathione peroxidase activity, and improved pathological changes in kidney and pancreas, likely through antioxidant effects and restoration of redox homeostasis [159].

Human field studies on forest bathing have provided compelling in vivo evidence of phytoncides’ hormonal and autonomic effects. Exposure to phytoncide-rich environments led to significant reductions in urinary cortisol, epinephrine, and norepinephrine, indicating decreased sympathetic nervous system activity. These physiological effects were supported by environmental measurements of forest air samples, which detected common phytoncides including α-pinene, β-pinene, and isoprene [160]. Supporting this evidence, even short-term forest stays resulted in significant decreases in serum cortisol, urinary adrenaline, testosterone, and blood pressure [161, 162]. These studies collectively suggest that phytoncide exposure can regulate stress-related endocrine markers through autonomic nervous system modulation.

The endocrine-modulating effects of phytoncides extend beyond natural outdoor environments. A randomized controlled trial (RCT) of 55 gynecological cancer survivors found that eight weeks (1 h/day, 5 days/week) of phytoncide fragrance exposure during meditation significantly reduced stress hormones (cortisol, epinephrine; *p* < 0.001) while increasing parasympathetic activity and NK cell levels, demonstrating coordinated endocrine-immune benefits [163].

#### Traditional applications

Traditional medicine systems employ a wide range of herbs—including ajwain (*Trachyspermum ammi*), *Aloe vera*, basil, coriander, ginseng, gurmar (*Gymnema sylvestre*), shatavari (*Asparagus racemosus)*, and oregano—to support endocrine health and treat conditions such as metabolic syndrome [164]. For female reproductive health, shatavari is specifically used to address hormonal imbalances. In vivo studies in female rats have shown that aqueous asparagus root extract (*Asparagus officinalis L.)* administration dose-dependently elevated hormones associated with the hypothalamic-pituitary-gonadal axis. The highest concentrations of GnRH, FSH, LH, estrogen, and progesterone were recorded at the maximum dose of 400 mg/kg. Additionally, the extract increased the number of ovarian follicles and corpus luteum, positively affecting oogenesis [165]. These herbs are traditionally administered orally, often as medicated decoctions, powders, or incorporated into the diet.

### Cardiovascular system

Phytoncides and EOs have emerged as promising natural agents for cardiovascular health, with evidence pointing to their roles in regulating blood pressure, lipid metabolism, and vascular inflammation. These effects are largely attributed to their rich composition of bioactive terpenes and phenylpropanoids—such as linalool, α-pinene, cineole, and cinnamaldehyde—which influence key physiological pathways involved in cardiovascular homeostasis [166].

One of the primary cardiovascular actions of EOs is vasorelaxation, contributing to their hypotensive effects. Many EOs act on vascular smooth muscle by modulating calcium channel activity and enhancing nitric oxide (NO) signaling. In vivo administration of java citronella (*Cymbopogon winterianus*) EO in Wistar rats induced dose-dependent hypotension accompanied by tachycardia at lower doses, while ex vivo studies using isolated rat mesenteric artery rings demonstrated endothelium-independent vasorelaxation through Ca²⁺-channel blockade [167]. Similarly, wild basil (*Ocimum gratissimum*) EO induced dose-dependent hypotension and bradycardia through direct vascular smooth muscle relaxation, with enhanced effects in hypertensive rats, effects that appear mediati promote vascular relaxation in vivo in Wistar rats [168, 169]. Shell ginger (*Alpinia zerumbet*) demonstrated endothelium-dependent vasorelaxation via the NO-cGMP pathway in ex vivo experiments using endothelium-intact rat aortic preparations [170, 171]. Similarly, *Seseli pallasi* EO, rich in α-pinene, exhibited potent vasorelaxant effects in ex vivo rat mesenteric artery rings, likely mediated through calcium channel blockage and NO release. Additionally, in vitro assays demonstrated dose-dependent angiotensin converting enzyme (ACE) inhibitory activity, with molecular docking studies identifying spathulenol as a key contributor. These findings suggest the potential of this EO to modulate hypertension through dual mechanisms: direct vascular effects and ACE inhibition within the renin-angiotensin-aldosterone system (RAAS) cascade [172].

Turmeric oil demonstrates cardiovascular protective effects. In hyperlipidemic hamsters, turmeric oil significantly redacted total cholesterol, LDL-cholesterol, and triglycerides while increasing HDL-cholesterol, accompanied by improved vascular function, reduced platelet activation, and decreased oxidative stress. These effects appear mediated through activation of PPARα and LXRα pathways [173].

Cinnamaldehyde, a key constituent of cinnamon oil, demonstrates antithrombotic properties. In vitro studies showed that cinnamaldehyde inhibited both collagen- and thrombin-induced platelet aggregation in rat platelets in a dose-dependent manner. In vivo, cinnamaldehyde administration prolonged hemorrhage and coagulation times, reduced mortality in collagen-epinephrine-induced pulmonary thromboembolism models, and decreased thrombus weight in arteriovenous shunt models in rodents, with effects comparable to aspirin [174].

However, higher doses may provoke transient bradycardia or arrhythmias due to parasympathetic activation, as observed with java citronella EO in rats. These effects were reversed by atropine or vagotomy, suggesting muscarinic receptor involvement [167].

#### Traditional applications

Traditional medicine systems utilize herbs such as ginseng, *Ginkgo biloba* [175], arjuna (*Terminalia arjuna*) [176], and jatamansi [177] for treatment of cardiovascular diseases. These herbs are typically administered orally as herbal decoctions or medicated milk. Recent research has validated these traditional uses. Advanced analytical approaches integrating GC/MS, network pharmacology, and ex vivo vascular activity testing in murine aortic rings identified β-maaliene and patchouli alcohol as bioactive constituents of jatamansi that exert vasodilatory effects by targeting NOS3 and PTGS2, supporting its potential for managing essential hypertension [177].

### Respiratory system

As volatile compounds, phytoncides naturally target the respiratory system through inhalation, where they exert antimicrobial effects against respiratory pathogens and influence pulmonary physiology. These effects have potential implications for both infectious disease control and chronic respiratory support.

The antimicrobial properties of EOs in vapor form have been particularly well documented. In vitro assays of 14 EOs, including cinnamon bark, lemongrass, and thyme, evaluated gaseous antibacterial activity against common respiratory pathogens such as *Haemophilus influenzae*, *Streptococcus pneumoniae*, *Streptococcus pyogenes*, and *Staphylococcus aureus*. Vapors from EOs rich in terpene alcohols and aldehydes showed the most potent effects, with *H. influenzae* being most sensitive. Antibacterial efficacy was greatest when high vapor concentrations delivered over short periods, indicating that delivery method and timing are critical factors [178].

Supporting these findings, in vitro studies of geranium and lemongrass EO blend demonstrated broad-spectrum activity against both antibiotic-sensitive and -resistant bacterial strains, including MRSA and vancomycin-resistant *Enterococci* (VRE). In situ evaluation using a sealed-box setup showed that EO vapors significantly reduced both airborne and surface bacterial loads when dispersed via a controlled vaporizing system. In practical applications, continuous exposure to EO vapors over 15 h in an office environment led to an 89% reduction in airborne bacteria, highlighting their practical potential for enhancing indoor air quality and limiting microbial transmission [179].

Beyond antibacterial activity, EOs also exhibit antiviral activity. Aerosolized tea tree oil and eucalyptus oil reduced airborne Influenza A virus levels by over 95% within 5–15 min in vitro, with tea tree oil inactivating over 99% of the virus. This effects persisted with prolonged vapor exposure, suggesting EO vapors may serve as natural disinfectants against airborne respiratory viruses—an application of particular relevance during seasonal flu outbreaks or viral pandemics [180].

The physiological impact of plant volatiles on respiratory function has been demonstrated through in vivo and in vitro studies. A randomized, controlled, open-label trial measured pulmonary function before and after forest bathing compared to urban exposure. Those who walked in a forest environment demonstrated significant improvements in both forced expiratory volume in 1 s (FEV1) and FEV6, standard measures of lung capacity. These improvements suggest a potential bronchodilatory or airway-soothing effect of forest air, likely mediated by inhaled phytoncides [181]. In vitro studies have elucidated specific anti-inflammatory pathways. In human monocytes, 1,8-cineole suppressed pro-inflammatory cytokines (TNF-a, Il-1b) and arachidonic acid metabolites (leukotriene B4 and thromboxane B2) in a dose-dependent manner, positioning 1,8-cineole as a potential treatment for airway inflammation in chronic obstructive pulmonary disease (COPD) [151].

Clinical trials support these findings. In a randomized, double-blind, placebo-controlled trial, 32 patients with steroid-dependent bronchial asthma received 200 mg of 1,8-cineol three times daily during progressive oral glucocorticosteroid (GCS) reduction every 3 weeks. The 1,8-cineol group tolerated 36% GCS reductions versus 7% with placebo, indicating significant GCS-sparing capacity [182]. Inhalation of cedrol, a component of cedarwood oil, significantly reduced respiratory rate, heart rate, and blood pressure in clinical studies through baroreceptor sensitivity and parasympathetic activation [183].

#### Traditional applications

Traditional medicine systems utilize diverse herbs such as tulsi (*Ocimum sanctum*), vasa (*Adhatoda Vasica*), peppermint, thyme, licorice, and aromatic spices like anise, and ginger for respiratory health [184]. These botanicals are commonly administered through diet, as medicated decoctions, or through therapeutic vapor inhalation. Spices are typically used for their immunomodulatory and antimicrobial properties, while herbs offer bronchodilator and expectorant effects. Multi-herb formulations are often designed to target the diverse pathophysiological features of respiratory diseases.

Recent research validates these traditional uses. Compounds in tulsi leaf extract (eugenol, cyclohexane, and caryophyllene) downregulated oxidative stress markers, including ROS, total oxidants, malondialdehyde (MDA), and myeloperoxidase (MPO), while significantly increasing total antioxidant capacity and SOD, CAT and GPx in a COPD mouse model. Molecular docking confirmed strong binding interactions with antioxidant enzymes, supporting tulsi’s potential in COPD treatment [185].

### Integumentary system

Phytoncides have demonstrated promising effects on skin health through their antimicrobial, anti-inflammatory, and antioxidant actions. As the skin serves as the body’s primary interface with the external environment, it is particularly vulnerable to oxidative damage, microbial invasion, and immune dysregulation. Evidence suggests that EOs can interact with these biological pathways to support and protect integumentary function.

Citrus EOs demonstrated notable antibacterial activity against skin pathogens *Propionibacterium acnes* and *Staphylococcus epidermidis*, with anti-inflammatory effects mediated through reduction of TNF-α and IL-8 production. The antimicrobial activity involves multiple components including limonene, α-terpineol, and linalool [186]. A comprehensive review of over 90 commercially available EOs for dermatological use found that dermatophytes were most sensitive to EO inhibition, followed by *Candida albincans* and Gram-positive bacteria, with Gram negative bacteria being most resistant. When EOs are combined with conventional antimicrobials, interactions are highly pathogen-specific, ranging from synergistic to antagonistic depending on specific combination [187]. The increasing prevalence of antibiotic-resistant skin infections underscores the potential value of EOs as alternative or complementary antimicrobial agents.

Beyond infection control, some EOs also promote wound healing and skin regeneration in burns, ulcers, and other dermal injuries. In diabetic rat wound models, Curry plant (*Helichrysum italicum)* EO accelerated wound contraction, increased hydroxyproline/collagen content, and promoted tissue regeneration with restitution of adnexal structures (188). In dynamic in vitro models mimicking skin physiology, *H. italicum* hydrolate stimulated collagen production and activated stemness gene expression in skin stem cells, supporting its regeneration potential [189].

Mechanistically, ex vivo studies with rat abdominal skin demonstrated that EOs can enhance skin permeability by disrupting the lipid architecture of the stratum corneum. This property has led to their investigation as penetration enhancers in transdermal drug delivery. Using attenuated total reflectance-Fourier transform infrared spectroscopy (ATR-FTIR), researchers observed that cinnamon and chuanxiong oils increased the fluidity and disorder of skin lipids, facilitating percutaneous absorption with enhancement ratios of 2.63 and 2.60, respectively—values exceeding the standard penetration enhancer azone. In vitro viability assays confirmed that these effects occur without significant cytotoxicity in HaCaT keratinocytes [190].

The antioxidative potential of lemon EO was demonstrated in an RCT of 80 male volunteers evaluating topical application following daily UV exposure for seven days. The oil effectively scavenged UV-induced ROS such as superoxide anions and peroxyl radicals in skin tissue. At 1:100 dilution in DMSO or grape-seed oil, lemon EO demonstrated antioxidant activity exceeding α-tocopherol (vitamin E) against superoxide anions and peroxyl radicals, indicating potent protection against photoaging and oxidative skin damage [191]. However, the potential for some EO constituents to cause irritation or sensitization underscores the need for concentration- and context-dependent evaluation.

#### Traditional applications

Traditional medicine systems have long used the skin as a primary route for drug delivery through massage, herbal pastes, ointments, and heat therapies, promoting both local and systemic healing. These practices leverage the skin’s absorptive capacity to deliver medicinal compounds and trigger systemic physiological responses. Herbs such as turmeric, sandalwood, and *Aloe vera* are traditionally used to treat various skin conditions due to their antimicrobial, anti-inflammatory, and wound-healing properties [192]. For example, curcumin has suppressed excessive TNF-α production by activated macrophages, reduced keratinocyte transferrin receptor expression, alleviated parakeratosis severity, and lowered epidermal CD8 + T-cell density in in vivo and in vitro studies, demonstrating efficacy for psoriasis treatment [193].

### Mental health benefits

EOs have demonstrated anxiolytic, antidepressant, and psychostimulant properties.Their neurophysiological effects are largely mediated by key constituents, such as linalool, limonene, myrtenol, and α-pinene that interact with neurochemical pathways central to mood and emotional regulation, notably the GABAergic, serotonergic, and dopaminergic systems [194]. Enhanced signalling of the GABA_A_ receptor, the brain’s main inhibitory mechanism, contributes to anxiolytic effects and improved emotional stability. Additionally, these compounds influence serotonergic modulation, a key target in depression and anxiety treatment [129].

In a pilot study at a mental health treatment center with 57 participants, bergamot EO (BEO) inhalation improved positive affect scores, with participants exposed to the EO reporting a 17% higher positive mood compared to controls (though not statistically significant). More participants enrolled in the BEO group (*n* = 45) versus control (*n* = 12), potentially indicating greater willingness to participate when exposed to the aroma [195]. Mechanistically, BEO appears to act as a psychostimulant, influencing ascending neurotransmitter systems involved in arousal, including serotonergic, cholinergic, noradrenergic, and histaminergic pathways. In animal models, systemic BEO administration increased exploratory behavior and elevated fast-frequency EEG activity in the hippocampus and cortex, suggesting enhanced alertness and cognitive engagement [196].

These results are consistent with human forest exposure studies, where participants walking through phytoncide-rich environments led to improved Profile of Mood States scores. Forest exposure consistently reduced negative mood indicators, including “tension-anxiety,” “confusion,” and “anger-hostility” [198].

#### Traditional applications

Ashwagandha, brahmi, ginkgo, kava (*Piper methysticum*), passion flower, and chamomile are among the key herbs traditionally used to support mental health, particularly for anxiety disorders [200]. In Ayurvedic practice, ashwagandha is administered orally as root extracts or decoctions and function as adaptogens that modulate the hypothalamic-pituitary-adrenal (HPA) axis, contributing to stress reduction and exerting anxiolytic effects on the CNS [201].

**Table 1:** Summary of influence of Plant-derived compounds on key human organ systems

Common name	Binomial name	Key phytoncides	Study type	Biological activity	Molecular targets and pathways	Refs.
**Immune System**						
Pine trees & other conifers		α-Pinene	In Vitro; In Vivo	Immunomodulatory; NK cell activation; Anticancer	Increases CD56, CD107a (NK cell surface markers); Increases perforin, granzyme B (cytolytic effectors); Activates ERK/AKT signaling pathways	[114]
Forest Phytoncides		α-Pinene; β-Pinene	Clinical Trial; In Vitro	NK cell activation; Anticancer; Stress reduction	Increases NK cell activity, CD16 + NK cell numbers, perforin, granulysin, granzymes A/B-expressing lymphocytes; Decreases urinary adrenaline and noradrenaline (stress-hormones); No effect on T cell numbers	[62, 115]
Coniferous trees; Rosemary		α-Pinene	Clinical Trial; In Vitro	Anti-inflammatory	Inhibits MAPK and NF-κB pathways; Reduces IL-6, TNF-α, NO production, Decreases iNOS and COX-2 expression in LPS-stimulated macrophages	[9]
Lavender; Coriander/Cilantro	*Lavandula augustifolia; Coriandrum sativum L (Plantaginaceae)*	Linalool	In Vitro	Anticancer; Immunomodulatory	Induce apoptosis in cancer cells (increased sub-G1 phase, dose-dependent); Inhibits T-47D cell migration; Stimulates Th1 cytokines (IFN-γ, IL-2, IL-13, TNF-α, CD40)	[116]
			In Vivo	Antimicrobial; Anti-inflammatory; Wound healing	Reduces inflammatory cytokines (IL-6, IL-10, IL-17, IL-23) in MRSA-infected mice; Decreases inflammatory cell infiltration (primarily neutrophils); Promotes fibroplasia and neovascularization)	[117]
		Linalool; Linalyl acetate	In Vivo	Anti-inflammatory	Reduces carrageenin-induced paw edema; Mechanism not specified	[118]
Citrus fruits	*Citrus* spp.	d-Limonene	Ex Vivo	Immunosuppressive; Cytokine inhibition; T cell modulation	Inhibits Th1 cytokines (IFN-γ, IL-2, TNF-α) and Th2 cytokines (IL-4, IL-13) by CD3+, CD4+, CD8 + T cells; Downregulates activation markers (CD25, CD69, CD40L)	[119]
Eucalyptus	*Eucalyptus* spp.	1,8-Cineole	In Vivo	Dose-dependent immunomodulation; Low-moderate dose: immune enhancement; High doses: immunosuppressive	Low-moderate doses (30–100 mg/kg): upregulates CD8 + T-cells in bronchoalveolar lavage fluid; No effect on macrophage phagocytosis or CD4 + cells; High dose (300 mg/kg): suppresses CD8 + T-cells, inhibits macrophage phagocytosis, reduces blood B cells, NK cells, decreases IgA (100–300 mg/kg)	[120]
Oregano	*Origanum vulgare*	Carvacrol	In Vivo	Anti-inflammatory	Modulates TLR/NF-κB pathway; Reduces gene expression of TNF-α, IL-1β, IL-6, TLR4, NF-κB p65, AVBD-9	[121]
Thyme	*Thymus vulgaris*					
Cinnamon	*Cinnamomum* spp.	β-Caryophyllene (BCP)	Review of In Vivo and In Vitro studies	Anti-inflammatory; Antioxidant; Antimicrobial; Immunomodulatory	Activates CB2 and PPARγ pathways; Suppresses IL-6 protein and mRNA expression; Reduces pro-inflammatory cytokines; Increases anti-inflammatory cytokine IL-13; Activates of Nrf2/HO-1 antioxidant axis; Inhibits HMG-CoA reductase activity; Reduces oxidation stress biomarkers	[122]
Black pepper	*Piper nigrum*					
Clove	*Syzygium aromaticum*					
Rosemary	*Rosmarinus officinalis*					
Ginger	*Zingiber officinale*	6-Gingerol	In Vitro & In Vivo	Anti-inflammatory	NF-kappa B and PKC signaling; anti- tumor properties linked to pathways suppressing lung cell metastasis	[123–125]
**Neurological system**						
**Cognitive Function**						
Peppermint	*Mentha piperita*	Menthol; Menthone	In Vitro Clinical Trial	Enhances synaptic activity; Enhances memory, sustained attention and reduced mental fatigue	Inhibit acetylcholinesterase and interacts with GABA_A_ and nicotinic receptors, increasing calcium mobilization in neurons	[126]
Sandarac/Arar tree	*Tetraclinis articulata*	Monoterpene hydrocarbons	In Vivo & In Vitro	Cognitive stimulation, antioxidant activity	Restore hippocampal acetylcholinesterase activity and antioxidant balance	[130]
Lemon	*Citrus limonum*	d-Limonene	Clinical trial	Faster reaction times but reduced inhibitory control and memory sensitivity		[131]
Brahmi	*Bacopa monnieri*	Triterpenoid saponins (e.g., bacosides)	In Vivo	Cognitive function	Restore synaptic activity; upregulate neuronal synthesis, kinase activity; increase secretion and availability of neurotransmitters like serotonin.	[133]
**Microbiome and the Gut-Brain Axis**						
Eucalyptus	*Eucalyptus globulus*	1,8-Cineole	In Vivo	Sedative-hypnotic; Sleep-promoting; Gut microbiota modulation	Increase sleep promoting neurotransmitters (GABA, glutamine, glycine, tryptophan, N-acetylserotonin, 5-HIAA); Enriches GABA- and glycine-synthesizing gut microbes; Increase SCFA-producing microbiota; Increases gut microbial diversity (Shannon, ACE, Chao1 indices); Increases Firmicutes, Proteobacteria, Actinobacteria; Decreases Bacteroidota/Firmicutes ratio	[136]
May chang	*Litsea cubeba*	Citral	In Vivo; In Silico	Anti-inflammatory; Gut protective; Anti-diarrheal; Gut microbiota modulation	Citral binds IL-1β, IL6, TNF-α via hydrogen bonds; Reduces hepatic and intestinal TNF-α, IL-6, and IL-1β levels; Protects colonic crypts and epithelial integrity; Increases *Lactobacillaceae*,* Lachnospiraceae*,* Eggerthellaceae*,* Marinifilaceae;* Decreases *Muribaculaceae;* Negative correlation with inflammation: *Lactobacillaceae*,* Bifidobacteriaceae*	[137]
Turmeric	*Curcumin longa*	Curcumin	In Vivo; Review	Neuroprotective; Anti-inflammatory; Gut microbiota modulation	Increases tyrosine hydroxylase-positive neurons; Elevates dopamine; Inhibits α-synuclein aggregation; Reduces TNF-α, IL-1β, IL-6; Reduces glial activation; Decreases N-acetylneuraminate degradation; Gut microbiota-dependent neuroprotection	[138–140]
Oregano	*Origanum vulgare*	Carvacrol	In Vitro	Selective antimicrobial; Pathogen suppression; Probiotic-sparing	Inhibits pathogenic bacteria (*C. perfringens*,* S. epidermine*,* E. coli*,* Salmonella serovars)* while moderately affecting or sparing beneficial bacteria (*B. breve*,* B. longum*,* L. reuteri*)	[141]
Thyme	*Thymus vulgaris*	Thymol				
Cinnamon	*Cinnamommum verum*	Cinnamaldehyde				
Rosemary oil	*Rosmarinus officinalis*	1,8-Cineole				
**Pain Modulation**						
Eucalyptus	*Eucalyptus citriodora; E. tereticornis; E. globulus*	1,8-Cineole	In Vivo	Analgesic (peripheral, central); Anti-inflammatory	Hypothesized inhibition of the arachidonic acid metabolism (reduces prostaglandins and thromboxane); Inhibits carrageenan- and dextran-induced paw edema; Reduces neutrophil migration; Decreases vascular permeability	[147]
Black pepper	*Piper nigrum*	β-Caryophyllene	Clinical Trial; In Vivo	Analgesic; Anti-inflammatory	Selective CB2 receptor agonist; Reduces inflammatory pain	[148, 149]
Lavender, Marjoram, Clary sage	*Lavandula officinalis* *Origanum majorana* *Salvia sclarea*	Linalyl acetate; Linalool; 1,8-Cineole	Clinical Trial	Analgesic (dysmenorrhea); Reduces menstrual pain duration	Inhibits TNF-α, IL-1β, leukotriene B4, thromboxane B2; Mechanisms for other components in dysmenorrhea not established	[150, 151]
Lavender oil	*Lavandula officinalis*	Linalool	Clinical Trial	Analgesic; Opioid-sparing	Mechanism not investigated; Reduced post-operative opioid requirements; Less sedation at discharge	[152]
Camphor laurel tree	*Cinnamomum camphora*	Camphor	In Vitro	Analgesic; Counterirritant	Activates and rapidly desensitizes TRPV1 (more complete desensitization than capsaicin); Inhibits TRPA1; Activation sites distinct from capsaicin; Enhanced by heat, PKC activation, and PLC-coupled receptor stimulation	[154]
Frankincense/Shallaki	*Boswellia serrata*	3-O-Acetyl-11-keto-β-boswellic acid	Meta Analysis	Analgesic; Anti-inflammatory; Anti-arthritic	AKBA inhibits 5-LOX and leukotriene synthesis	[156]
**Endocrine System**						
Ylang-Ylang	*Cananga odorata*	Benzyl salicylate	In Vitro	Hormonal regulation	Increases hPL	[157]
Niaouli	*Melaleuca quinquennervia*	1,8-Cineole			Increases progesterone, hCG, hPL	
Wintergreen	*Gaultheria procumbens*	Methyl salicylate			Increases progesterone, hCG, hPL; Whole oil effects opposite to isolated methyl salicylate	
Orange	*Citrus sinensis*	Limonene			Increases estradiol, hCG, hPL; Whole oil effects opposite to isolated limonen	
Tea tree	*Melaleuca alternifolia*	4-Terpineol			Increases hPL; Whole oil effects differ from isolated 4-terpineol	
Cinnamon	*Cinnamomum verum*	Eugenol; Cinnamaldehyde	In Vitro; In vivo	Improves insulin sensitivity; Reduces blood glucose; Enhances glucose tolerance; Reduces hepatic steatosis; Increases liver lycogen	Enhances insulin signaling via GSK3 and AKT phosphorylation (in astrocytes); Improves brain insulin sensitivity; Modulates brain-liver axis via STAT3 signaling; No direct hepatic effects (mediated centrally)	[158]
Fennel	*Foeniculum vulgare*	Anethole; Fenchone; Estragole	In Vivo	Hypoglycemic; Antioxidant; Nephroprotective; Pancreatic protection	Enhances glutathione peroxidase activity; restores redox homeostasis	[159]
Asparagus	*Asparagus officinalis L.*	Saponins	In Vivo	Hormonal regulation; Promotes oogenesis	Stimulates hypothalamic-pituitary-gonadal axis; Increases GnRH, FSH, LH, estrogen, progesterone (dose-dependent); Increases number of primordial, primary, and graafian follicles; Increases corpus luteum	[165]
**Cardiovascular System**						
Java citronella	*Cymbopogon winterianus*	Citronellol; Citral; Linalool	In Vivo; Ex Vivo	Hypotensive; Vasorelaxation; Dose-dependent effects on heart rate (lower doses: tachycardia; high dose: transient bradycardia and arrhythmias)	Ca²⁺-channel blockade (endothelium-independent vasorelaxation); Muscarinic receptor activation (cardiac effects at high dose mediated by vagal discharge); partially mediated by cholinergic pathways	[167]
Wild basil	*Ocimum gratissimum*	Eugenol; 1,8-Cineole; Linalool; Thymol; Geraniol	In Vivo	Hypotensive; Bradycardic; Vasorelaxation	Direct vascular smooth muscle relaxation (independent of the autonomic nervous system); bradycardia mediated by autonomic pathways (vagal/cholinergic); hypotension independent of autonomic control	[168, 169]
Shell ginger	*Alpinia zerumbet*	1,8-Cineole; Terpinene	Ex Vivo	Vasorelaxation; Endothelium-dependent vasodilation	Nitric oxide-cGMP pathway; requires functional endothelium; Independent of prostacyclin and β-adrenoceptors	[170, 171]
Seseli pallasi	*Seseli pallasi*	α-Pinene	Ex Vivo; In Vitro; In Silico	Vasorelaxation; ACE inhibition; Antihypertensive	Inhibits voltage-dependent Ca²⁺ channels; Enhances NO/cGMP pathway; Inhibits ACE; Sapthulenol shows high binding affinity to ACE	[172]
Turmeric	*Curcuma longa*	Curcumin	In Vivo	Anti-hyperlipidemic; Improves vascular function; Anti-platelet Antioxidant	Activates PPARα and LXRα; Modulates genes involved in cholesterol metabolism (CYP7A1, ABCA1, ABCG5/G8, LPL); Suppresses SREBP-2 and HMGCR	[173]
Cinnamon	*Cinnamomum verum*	Anethole; Cinnamaldehyde	In Vitro	Antiplatelet; Antithrombotic	Inhibits collagen- and thrombin-induced platelet aggregation; Reduces thrombus formation; Mechanism of platelet inhibition unclear	[174]
Jatamansi	*Nardostachys jatamansi*	Valerena-4,7(11)-diene; Calarene; β-Maaliene; Patchouli alcohol	Ex Vivo; Network Pharmacology	Vasodilatory; Antihypertensive	Targets NOS3 and PTGS2; Modulates lipid and atherosclerosis pathways; Affects PI3K-AKT signaling	[177]
**Respiratory System**						
Cinnamon	*Cinnamomum verum*	Cinnamaldehyde; Eugenol	In Vitro	Antibacterial	Direct antimicrobial action; most active compounds: terpene alcohols and aldehydes	[178]
Lemongrass	*Cymbopogon citratus*	Citral; Geraniol; Neral				
Thyme	*Thymus vulgaris*	Thymol; Carvacrol				
Geranium; Lemongrass	*Pelargonium × hortorum*	Citronellal; Geraniol; Linalool; Citral	In Vitro; In Situ	Antibacterial	Direct antimicrobial action against airborne and surface bacteria	[179]
	*Cymbopogon citratus*					
Tea Tree; Eucalyptus	*Melaleuca alternifolia*	Terpinen-4-ol; Terpinolene	In Vitro	Antiviral	Direct viral inactivation	[180]
	*Eucalyptus globulus*	1,8-Cineole				
Eucalyptus	*Eucalyptus globulus*	1,8-Cineole	In Vitro; Clinical Trial	Anti-inflammatory; Bronchodilatory; Glucocorticosteroid-sparing; Mucolytic	Inhibits pro-inflammatory cytokines (TNF-α, IL-1β); Suppresses arachidonic acid metabolites (leukotriene B4 and thromboxane B2); dose-dependent inhibition in LPS- and IL-1β-stimulated monocytes	[151, 181, 182]
Cedarwood	*Cedrus deodara*	Cedrol	Clinical Trial	Reduces respiratory rate, heart rate, and blood pressure; Anxiolytic	Enhances baroreceptor sensitivity; Increases parasympathetic activity (increased HF component of HRV); Reduces sympathetic activity (decreased LF/HF ratio, reduced vasomotor sympathetic activity); Autonomic modulation	[183]
Tulsi (Holy basil)	*Ocimum sanctum*	Eugenol; Cyclohexane; Caryophyllene	In Vivo; In Silico	Anti-inflammatory; Antioxidant	Downregulates ROS, total oxidants, MDA, MPO; Increases total antioxidant capactity and activity of key enzymes such as SOD, CAT & GPx; Strong binding affinity to antioxidant enzymes (molecular docking)	[185]
**Integumentary system**						
Geumgamja; Cheonyahagyul	*Citrus obovoides; Citrus natsudaidai*	Limonene; γ-terpinene	In Vitro	Antibacterial; Antioxidant; Anti-inflammatory	Inhibits *P.acnes* and *S. epidermidis growth; Reduces TNF-*α and IL-8 production; Scavenges superoxide radicals	[186]
90 + commercially available EOs			Review	Broad antimicrobial spectrum; Pathogen sensitivity	Lipophilic membrane disruption; Synergistic to antagonistic interactions with conventional antimicrobials (pathogen-specific)	[187]
Curry plant	*Helichrysum arenarium*	γ-Curcumene; Neryl acetate; α-Pinene	In Vivo; In Vitro	Wound healing; Promotes tissue regeneration	Accerlerates wound contraction; Increases total hydroxyproline content; Stimulates collagen production; Activates molecular program of stemness in skin stem cells; Improves redox status	[188, 189]
Cinnamon	*Cinnamommum verum*	*(E)-Cinnamaldehyde*	In Vitro, Ex Vivo	Transdermal penetration enhancement; Disrupts stratum corneum lipid barrier	Increases fluidity and disorder of SC lipids	[190]
Chuanxiong	*Ligusticum striatum*	*Ligustilide*				
Lemon	*Citrus limonum*	*Limonene*	Clinical trial	Antioxidant; Free radical scavenging; Photoprotective	Scavenges superoxide anions and peroxyl radicals; Prevents UV-induced lipid peroxidation	[191]
Turmeric	*Curcuma longa*	*Curcumin*	In Vitro; In Vivo; Clinical Trial	Anti-inflammatory; Reduces keratinocyte hyperproliferation	Suppresses TNF-α production by macrophages; Binds TNF-α receptor sites blocking NF-κB activation; Inhibits phosphorylase kinase; Reduces keratinocyte TRR expression, parakeratosis severity, epidermal CD8 + T-cell density	[193]
**Mental Health**						
Lavender; Bergamot; Rosemary; Ylang-ylang; Cinnamon	*Lavandula officinalis; Citrus bergamia; Rosmarinus officinalis; Cananga odorata; Cinnamommum verum*	Linalool; 1,8-cineole; limonene cinnamaldehyde	Review of In Vitro, In Vivo, Clinical Trial	Anxiolytic; Antidepressant; Psychostimulant	Enhances GABA_A_ receptor signaling; Modulates serotonergic system; Activates dopaminergic system; Inhibits neuronal voltage-gated Na + channels; Modulates HPA axis (decreases cortisol/corticosterone); Downregulates NF-κB	[129, 194]
Bergamot	*Citrus bergamia*	Linalool; Linalyl Acetate; Limonene	Clinical Trial; In Vivo	Psychostimulant; Improves positive affect; Increases alertness	Modulates serotonergic, cholinergic, noradrenergic, histaminergic pathways; Increases fast-frequency EEG activity (hippocampus, cortex); Dose-dependent increase in locomotor/exploratory behavior	[195, 196]
Ashwagandha	*Withania somnifera*	Withanolides	Review of Clinical and Preclinical Studies	Anxiolytic; Antidepressant; Adaptogenic; Neuroprotective; Anti-inflammatory; Immunomodulatory	GABA-mimetic activtiy (27× greater affinity for GABAρ1 vs. GABAA receptors); Modulates HPA axis; inhibits NF-κB, JAK/STAT, activator protein 1 (AP-1) pathways; activates Nuclear factor erythroid 2-related factor 2 (Nrf2); Inhibits stress-activated c-Jun N-terminal protein kinase (JNK1) and iNOS; modulates heat shock protein 70 (Hsp70); Reduces pro-inflammatory cytokines (IL-1β, IL-6, TNF-α); Exhibits AChE inhibition	[201]

### Adverse effects

Despite the discussed benefits, EO-based therapies are not without risk. A growing body of literature highlights that adverse effects—ranging from mild skin reactions to life-threatening toxicities—can occur, particularly with inappropriate use. A comprehensive review article of 42 case reports documented 71 instances of adverse reactions linked to aromatherapy. The most frequently reported effects were dermatological, such as contact dermatitis, often associated with lavender, tea tree, peppermint, bergamot, and ylang-ylang oils. More severe outcomes included seizures, coma, respiratory distress, and even death, with some cases requiring hospitalization or surgical intervention. These findings challenge the common perception that “natural” equates to “safe,” especially for vulnerable populations like children, pregnant women, or those with allergies [202].

EO toxicity is dose- and route-dependent, with hypersensitive individuals at particular risk regardless of dose. Improper storage can lead to the formation of toxic oxidation products, further increasing risk. Documented cases of poisoning from oils like pennyroyal, wormwood, cinnamon, and nutmeg have been reported in humans [203]. Even compounds with anti-inflammatory effects, like 1,8-cineole, may exert immunosuppressive effects at high doses, as demonstrated through in vivo animal models [120].

These findings highlight the need for rigorous safety assessments, regulatory oversight, and user education to minimize harm from essential oil exposure.

### Limitations and gaps in the current evidence

Several limitations constrain interpretation of the current evidence base. The predominance of preclinical data represents a fundamental constraint. While in vitroand animal studies establish biological plausibility and elucidate mechanisms, human clinical validation remains limited. The few available clinical trials often suffer from small sample sizes (*n* < 100), inadequate statistical power, and methodological weaknesses including insufficient binding and high attrition rates.

EO composition presents another challenge. Substantial variability arises from botanical source, geographic origin, cultivation practices, and extraction methods, yet comprehensive chemical characterization remains absent from many studies. This heterogeneity undermines reproducibility and clinical standardization. Notably, research demonstrating that whole oils produce effects distinct from their isolated constituents [157] underscores our incomplete understanding of synergistic interactions within complex botanical mixtures.

The gut-brain axis emerges as compelling mechanistic framework, particularly for neurological and metabolic effects, but evidence derives predominantly from animal models. Human microbiome studies are scarce, and the distinction between correlation and causation remains poorly defined. Forest bathing studies [62, 115], while suggestive of phytoncide-mediated effects, cannot isolate phytoncide effects from confounders (exercise, stress reduction, visual exposure). Similarly, many proposed mechanisms remain speculative rather than definitively established. Additionally, long-term safety data are limited, and potential adverse effects including sensitization, drug interactions, endocrine disruption, and effects during pregnancy use require systemic investigation. Route of administration significantly influences bioavailability yet varies across studies, complicating cross-study comparisons.

Future research should prioritize adequately powered, longitudinal human trials using standardized, chemically characterized preparations. Mechanistic studies must move beyond associations to establish causality. Systemic characterization of dose-response relationships, therapeutic windows, and individual variability—including genetic polymorphisms and baseline microbiome composition—will be essential. Finally, comparative effectiveness research will clarify whether phytochemicals represent viable alternative or complementary agents relative to complementary treatments.

## Outlook

### Bioengineering applications

Advances in bioengineering, wearable technologies, and environmental sensing are rapidly transforming our ability to characterize GxE with unprecedented resolution [204, 205]. Earlier approaches often relied on self-reported surveys, static satellite imagery or fixed-site environmental monitoring stations, which lacked the spatial and temporal granularity necessary to capture real-time, dynamic exposures. Additionally, these traditional methods failed to account for individual-level behavioral patterns such as commuting routes, occupational settings, and daily mobility, limiting their accuracy in estimating true personal exposures.

In contrast, personal exposure monitors (PEMs) offer individualized, high-resolution measurements of environmental factors, including temperature, humidity, UV radiation, noise levels, and particulate matter (PM2.5, PM10). Complementing this environmental monitoring, advanced technologies like next-generation sequencing and mass-spectrometry enable detailed quantification of chemical and biological exposures from collected biospecimens and environmental samples. Together, these approaches facilitate comprehensive, personalized exposure assessment across diverse indoor and outdoor environments [204].

Simultaneously, multi-omics technologies now enable comprehensive profiling of biospecimens such as peripheral blood, urine, hair, and stool, capturing a wide array of biomarkers. These include epigenetic modifications (e.g., DNA methylation, histone modifications), post-transcriptional regulation (e.g., alternative splicing, RNA modifications), proteomic and metabolomic signatures, and microbiome composition. These innovations offer mechanistic insight into how environmental exposures modify gene expression and downstream biological pathways [205, 206].

The widespread adoption of wearable devices—such as smartphones, smartwatches, rings, and continuous glucose monitors—now enables continuous tracking of individual physiological parameters, including sleep, cardiovascular, respiratory, and metabolic function, as well as physical activity and location via GPS. These data provide a dynamic window into how exposures may influence biological systems in near real-time, offering opportunities to explore GxE longitudinally [113]. When coupled with multi-omics profiling, these tools lay the foundation for developing personalized, plant-based therapeutic strategies that integrate environmental exposures, molecular signatures, and physiological responses.

### Translational considerations

#### Integration challenges and opportunities

Despite increasing scientific validation and public interest, substantial translational barriers remain for integrating phytochemicals into clinical practice. As mentioned in Sect. 2, current regulatory frameworks categorize phytochemicals as dietary supplements or cosmetics, and only rarely as botanical drugs, limiting their formal integration into clinical practice [46].

Moving forward, regulatory science will need to evolve beyond the conventional single-compound, single-target model to accommodate the unique characteristics of plant-based therapies. These preparations typically involve multi-compound mixtures with dynamic variability influenced by environmental and genetic factors [207]. New regulatory approaches that account for polypharmacology are essential for comprehensive safety and efficacy evaluations, enabling responsible translation of phytochemicals into precision medicine applications.

Additionally, plant-based compounds may offer complementary or alternative options alongside existing pharmaceutical and lifestyle interventions, potentially mitigating issues such as drug resistance or polypharmacy burdens. The National Center for Complementary and Integrative Health (NCCIH) refers to this as “complementary medicine” when used alongside conventional treatments, part of the broader framework of “traditional, complementary, and integrative medicine (TCIM).”

International health systems offer valuable precedents. Germany’s Commission E, India’s Ministry of AYUSH, and China’s integration of TCM in state hospitals demonstrate that botanical therapies, when accompanied by appropriate quality controls and safety standards, can coexist with allopathic medicine, be dispensed in pharmacies, and be covered by national insurance systems [208, 209]. These models demonstrate that, with appropriate oversight, natural compounds can be safely integrated into modern healthcare without compromising rigor or patient safety.

Beyond the clinical and regulatory integration, urban planning offers another translational opportunity by redesigning urban environments to include accessible green spaces, an approach that has demonstrated physiological and mental health benefits [210–212]. Studies such as the Green Heart Louisville project have shown that increasing urban green cover is associated with reduced inflammatory biomarkers and demonstrates beneficial effects on cardiovascular health, providing large-scale, population-level evidence for the health-promoting effects of neighborhood-integrated plant-based exposures [213, 214].

#### Technological innovation and novel trial designs

The convergence of big data, AI, and real-world evidence presents a transformative opportunity for healthcare. AI systems leverage complex, high-dimensional datasets to uncover patterns, predict outcomes, and enable personalized, holistic insights. While healthcare often operates with specialized disciplines and targeted interventions, AI can facilitate more integrative, systems-level analysis that better reflects the interconnected nature of human health. This approach mirrors the systems-based philosophy of traditional medicine, which emphasizes whole-person care and the restoration of homeostasis.

Despite advances in computational biology, wearable technology, multi-omics profiling, and digital therapeutics, many clinical and regulatory processes remain anchored in outdated frameworks, relying heavily on large, fixed-cohort RCTs, which are slow, expensive, and often misaligned with the heterogeneity of human biology. The standard drug development timeline still spans a decade or more [215]—a delay that is particularly unjustifiable for compounds with centuries of empirical use.

To accelerate integration, the biomedical ecosystem must embrace novel clinical trial designs, such as adaptive trials [216], N-of-1 designs, and pragmatic real-world trials [215], which offer flexible, patient-centric alternatives to traditional RCTs. These models allow for iterative refinement of interventions, incorporate ecological validity, and can stratify responders by genetic, metabolic, or microbiome profiles. Wearable sensors and mobile health platforms further enable low-burden, continuous outcome tracking, capturing sleep, mood, pain, inflammation, and autonomic responses outside laboratory settings. These technologies can be integrated into clinical trials to provide real-world evidence.

In the era of precision medicine, integration should no longer be delayed by outdated paradigms. Data-driven decision making, participatory research models, and interoperable digital tools can forge a new path for validating nature-based therapies as evidence-supported complements to biomedical care.

#### Standardization and harmonization

The standardization of phytochemical research workflows is essential to ensure rigor, reproducibility, and regulatory readiness. This spans authentication of plant materials, extraction methods, as well as chemical and biological characterization.

Botanical authentication includes the use of voucher specimens, DNA barcoding, and geo-tagged metadata to verify plant identity and origin. Standardized extraction protocols (e.g., hydrodistillation, supercritical CO₂ extraction) should clearly specify solvent types, temperatures, durations, and extraction yields to facilitate comparability across studies. Additionally, researchers should report yield percentages, GC/LC-MS chromatograms, retention indices (RI), and relative abundances of active constituents to support reproducibility and compound-specific analysis.

Beyond chemical profiling, bioactivity assays must also follow standardized protocols, including validated cell lines, animal models, defined dose ranges, consistent endpoints (e.g., cytokine levels, gene expression), and appropriate controls. Despite the existence of harmonized international standards, such as those from the United States Pharmacopeia (USP Herbal Medicines Compendium) [217], the European Pharmacopoeia [218], and the International Organization for Standardization (ISO 19609, ISO 22590) [219], adoption remains limited in academic and preclinical research. Barriers include limited awareness of best practices, inherent variability in plant materials, and lack of standardized enforcement mechanisms across journals and funding agencies.

To accelerate global reproducibility, researchers should adhere to data reporting principles like FAIR (Findable, Accessible, Interoperable, Reusable) and deposit spectral data, raw results, and metadata in open-access platforms such as PubChem [220, 221], Metabolomics Workbench [222, 223], and GNPS (Global Natural Products Social Molecular Networking) [224, 225]. Such standardization will be critical for translating traditional botanical knowledge and novel phytochemical discoveries into clinically relevant, data-driven frameworks, enabling large-scale meta-analyses and facilitating AI-driven therapeutic discovery.

### Alignment with the united nations sustainable development goals

Harnessing plant-derived compounds and nature-based interventions supports multiple United Nations (UN) Sustainable Development Goals (SDGs).

SDG3: Good Health and Well-being [226] is addressed through the promotion of accessible, culturally appropriate, and holistic health solutions that complement conventional pharmaceutical approaches, especially in resource-limited settings. In many parts of the world where access to pharmaceuticals and advanced medical care may be limited, strengthening the scientific understanding of naturally-occurring bioactive compounds and disseminating this knowledge through public health education will empower individuals, families, and healthcare providers to adopt affordable and personalized plant-based interventions that would otherwise be inaccessible.

SDG 11: Sustainable Cities and Communities [226] is addressed by integrating green infrastructure and nature-based solutions into urban design. Increasing access to urban green spaces offers scalable and equitable pathways to improve both physical and mental health outcomes while enhancing environmental sustainability and climate resilience. As urbanization accelerates worldwide, embedding nature-based interventions into city planning represents a vital intersection between planetary health and public health [227].

### Plants, climate change, and urban spaces

The relationship between human health, plant biology, and the environment is deeply interconnected. Climate change threatens ecosystems globally while simultaneously altering plant biochemistry, shifting the availability, potency, and diversity of beneficial phytochemicals with downstream effects on human health [228]. Changes in temperature, precipitation patterns, atmospheric CO_2_ levels, and environmental stressors can modify plant secondary metabolism, potentially influencing the composition and concentration of health-promoting compounds [229, 230].

Preserving biodiversity and protecting ecosystems are therefore critical not only for environmental sustainability but also for safeguarding the human health benefits derived from plant-based exposures. Plants play an important role in mitigating climate change through carbon sequestration, temperature regulation, and air purification; consequently, integrating green spaces and nature-based infrastructure into urban environments enhances both public health and climate resilience [231, 232].

## Conclusion

Plant-derived phytochemicals, particularly phytoncides, demonstrate measurable effects on human health through GxE. Integrating traditional knowledge from TCM, Ayurveda, and other medical systems with modern multi-omics approaches can illuminate how these compounds influence systemic health across molecular layers. This review has demonstrated phytochemical effects across six major organ systems, with particularly robust evidence for immunomodulation (α-pinene, linalool), cardiovascular protection (EO-mediated vasorelaxation), and gut-brain axis modulation (curcumin, eucalyptus oil).

Critical gaps remain: the evidence base is predominantly preclinical, EO composition lacks standardization, and many proposed mechanisms remain correlative rather than causal. Translating these finding into clinical practice requires rigorous human trials using chemicals using chemically characterized preparations, mechanistic studies establishing causality, and investigation of individual variability including genetic and microbiome factors. Larger-scale clinical trials adopting novel trial designs are essential to establish comprehensive safety and efficacy profiles across diverse populations. Such evidence will be essential for integration into global health policy and precision medicine.

Global organizations like the WHO, the UN, and the NCCIH increasingly support TCIM practices, yet realizing this potential will require dedicated regulatory frameworks that address the multi-compound nature of phytochemical mixtures while maintaining standards for quality, safety, and efficacy. Emerging technologies, including AI, high-throughput omics platforms, and precision health analytics, now enable the molecular resolution needed to characterize complex biological effects and guide evidence-based applications.

Beyond individual therapeutics, understanding beneficial plant-environment interactions offers population-level opportunities through urban green space integration and nature-based solutions. As climate change threatens plant biochemistry and ecosystem stability, preserving biodiversity becomes critical not only for environmental sustainability but also for maintaining access to beneficial phytochemical exposures. Only through interdisciplinary collaboration spanning traditional medicine, rigorous clinical and translational research, regulatory science, and public health can phytochemical research advance from promising preclinical findings to validated interventions in precision public health and inform nature-based public health strategies.

## Data Availability

No datasets were generated or analysed during the current study.
